# A Phytochemical and Biological Characterization of *Cynara cardunculus* L. subsp. *scolymus* Cultivar “Carciofo di Procida”, a Traditional Italian Agri-Food Product (PAT) of the Campania Region

**DOI:** 10.3390/molecules30153285

**Published:** 2025-08-05

**Authors:** Giuseppina Tommonaro, Giulia De Simone, Carmine Iodice, Marco Allarà, Adele Cutignano

**Affiliations:** CNR-Istituto di Chimica Biomolecolare, Via Campi Flegrei, 34, 80078 Pozzuoli, Naples, Italy; giuseppina.tommonaro@cnr.it (G.T.); giuliadesimone@cnr.it (G.D.S.); carmine.iodice@cnr.it (C.I.); marco.allara@cnr.it (M.A.)

**Keywords:** Romanesco globe artichoke, polyphenols, cynarasaponins, anthocyanins, UHPLC-HRESIMS/MS, antioxidant activity, cytotoxic activity, CaCo-2 cell line, SH-SY5Y cell line

## Abstract

The artichoke (*Cynara cardunculus* L. subsp. *scolymus*) is an endemic perennial plant of the Mediterranean area commonly consumed as food. It is known since ancient times for its beneficial properties for human health, among which its antioxidant activity due to polyphenolics stands out. In the frame of our ongoing studies aiming to highlight the biodiversity and the chemodiversity of natural resources, we investigated the phenolic and saponin content of the cultivar “Carciofo di Procida” collected at Procida, an island of the Gulf of Naples (Italy). Along with the edible part of the immature flower, we included in our analyses the stem and the external bracts, generally discarded for food consuming or industrial preparations. The LCMS quali-quantitative profiling of polyphenols (including anthocyanins) and cynarasaponins of this cultivar is reported for the first time. In addition to antioxidant properties, we observed a significant cytotoxic activity due to extracts from external bracts against human neuroblastoma SH-SY5Y cell lines with 43% of cell viability, after 24 h from the treatment (50 μg/mL), and less potent but appreciable effects also against human colorectal adenocarcinoma CaCo-2 cells. This suggests that the different metabolite composition may be responsible for the bioactivity of extracts obtained from specific parts of artichoke and foresees a possible exploitation of the discarded material as a source of beneficial compounds.

## 1. Introduction

*Cynara cardunculus* L. subsp. *scolymus* (Asteraceae), commonly known as globe artichoke, constitutes a food plant whose interest is continuously increasing not only due to its nutritional value but also for a multiplicity of beneficial properties known since ancient time due to its high content of bioactive compounds, such as polyphenols and inulin [1,2,3]. Its estimated total world production for 2023 was 1,609,935 tons, with a 10% increase with respect to 2020 according to the FAOSTAT database (https://www.fao.org/faostat/en/#data/QCL (accessed on 11 July 2025)). From the Mediterranean area where it has been originally cultivated it has spread worldwide: Italy is one of the top producers with 369,720 tons per year, second only to Egypt (449,800 tons per year) and claims a long list of traditional food recipes. The edible part is represented by the immature inflorescence, but it has been also used in traditional medicine for its therapeutic effects, including antioxidant, hepatoprotective, cholesterol-lowering, hypoglycemic, choleretic, anticarcinogenic, and antibacterial properties [4,5,6,7,8,9,10,11,12,13].

The main compounds responsible for these effects are considered to be its polyphenols, particularly mono- and dicaffeoyl quinic acids which generally are among the most abundant constituents in the extracts. Other metabolites such as the flavonoids, apigenin and luteolin, and their glycosides have been also widely described in *Cynara* spp. with various relative abundances and being in some varieties the dominant metabolites [14]. Indeed, it is well known that the profile and the levels of artichoke secondary metabolites may greatly vary according to multiple factors such as the genotype and the cultivar, but also the climatic conditions, the soil characteristics, and the growth stage: phytochemical content is therefore a dynamic parameter since metabolic changes based on physiological and environmental conditions occur normally [15,16,17,18,19,20].

In the frame of our ongoing studies on bioactive metabolites from natural sources, with the aim of exploring the chemodiversity of terrestrial plants of nutritional and therapeutic interest, we investigated the polyphenol and triterpenoid saponin content of the cultivar “Carciofo di Procida” collected at Procida island, in the Gulf of Naples (Italy). This cultivar is a Roman-like artichoke producing large, globose primary flower heads that are light green with purplish tint, and it has been recently included by the Italian Ministry of Agriculture, Food Sovereignty and Forests in the list of PAT (Prodotti Agroalimentari Tradizionali, also referred as TAP, Traditional Agri-food Products) of Campania in Southern Italy (https://www.masaf.gov.it/flex/cm/pages/ServeBLOB.php/L/IT/IDPagina/22781 (accessed on 31 July 2025)). This recognition is attributed to products that for at least 25 years have been cultivated and/or processed according to traditional methods and refer to specific geographic areas [21]. Campania is one of the regions where suitable climatic and soil conditions have led to the selection of many landraces. The most famous and widely cultivated variety is the so-called “Carciofo di Paestum”, a Romanesco-type PGI (Protected Geographical Indication) product used as a base ingredient of many traditional foods and deeply investigated for its nutritional and health beneficial properties [14,17,22,23,24,25]. These studies applied NMR spectroscopy and/or mass spectrometric techniques to provide metabolomic fingerprinting of the extracts which were used to compare various landraces and selected clones; they evidenced large differences in polyphenol quali-quantitative profiles and in the antioxidant activity, as well [14,22,23,24,25,26]. The chemical characterization and bioactivity evaluation of the various cultivars increase the knowledge of the distinctive traits of agri-food products and may contribute to the valorization and selection of diverse varieties/clones in the territory of origin.

So far, the chemical profiling and the biological properties of the cultivar “Carciofo di Procida” have been never investigated. Here, we report the specific metabolite content in its different parts, namely the heart with inner bracts, which are typically consumed by both home-made and industrial food preparation, as well as external bracts and stem: the latter two account approximately for the 50% and 20%, respectively, of fresh commercial artichoke and are both reputed as waste material in some food preparations and canning processes. The biological evaluation of hydroalcoholic extracts for antioxidant and cytotoxic activities on four tumoral cell lines is also described.

## 2. Results

### 2.1. Evaluation of the Total Polyphenol Content (TPC)

The total polyphenolic content (TPC) of hydroalcoholic extracts of the heart with inner bracts (H), external bracts (E), and stem (S) of cultivar “Carciofo di Procida” was estimated according to the Folin–Ciocalteau method [27]. E exhibited the greatest TPC (3.4 ± 0.2 mg/g DW), followed by H (1.20 ± 0.06 mg/g DW) and S (0.75 ± 0.04 mg/g DW) (Table 1; Supplementary Material, Table S1 for data expressed as mg/g extract).

### 2.2. Evaluation of the Total Anthocyanin Content (TAC)

The total monomeric anthocyanin content (TAC) in the different tissues of “Carciofo di Procida” was estimated according to ref. [28]. E displayed the highest value of TAC, corresponding to 12.70 ± 0.02 mg/100 g DW, followed by H with a value of 1.50 ± 0.04 mg/100 g DW (Table 1). Anthocyanins were not detected in S.

### 2.3. Qualitative Profile of Polyphenols and Saponins in Cynara cardunculus L. subsp. scolymus Extracts by UHPLC-ESIMS^−^

To perform chemical profiling, six fresh specimens of artichoke were individually dissected into heart with inner bracts (H, 6 subsamples) and stems (S, 6 subsamples); the external leaves (E) from the six specimens were pooled, and all the samples were lyophilized. The dry material was successively grounded, and the raw powder was extracted with ethanol (EtOH)/H_2_O (1:1) at 38 °C as reported in Section 4.8.1. The raw plant extract was resuspended in methanol (MeOH)/H_2_O (1:1) and directly analyzed by Ultra High Performance Liquid Chromatography-High Resolution Electrospray Ionization Tandem Mass Spectrometry (UHPLC-HRESI-MS/MS) on a Polar C18 column as reported in Section 4.8.2.

**Figure 1:**
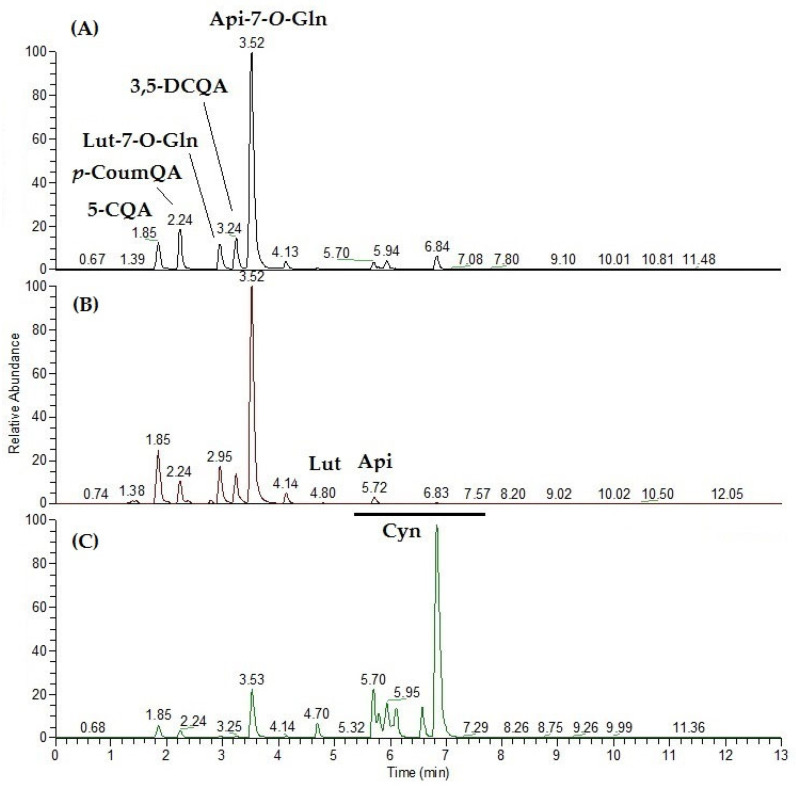
Representative UHPLC-ESIMS profiles in negative ionization mode (XIC—chromatograms by extracted [M−H]^−^ ion) of the hydroalcoholic extract of artichoke parts: (**A**) heart with inner bracts (H); (**B**) external bracts (E); and (**C**) stem (S). 5-CQA, chlorogenic acid; *p*-CoumQA, *p*-coumaroylquinic acid; Lut-7-*O*-Glu, Luteolin-7-*O*-glucoside; 3,5-DCQA, 3,5-dicaffeoyl quinic acid; Api-7-*O*-Gln, apigenin-7-*O*-glucuronide; Lut, luteolin; Api, apigenin; Cyn, cynarasaponins.

For polyphenol and saponin analyses, samples were analyzed in duplicate in negative ionization mode, and the LCMS profiles were compared (Figure 1). At a first glance, the molecular fingerprints of both H and E extracts were nearly overlapping. Several differences were indeed evident with respect to the S chemical profile. Main peaks were at first tentatively identified based on literature data and successively confirmed by running the corresponding commercial standards when available as reported in Figure 1. The most prominent peak in the artichoke head parts (H + E) is the flavonoid apigenin-7-*O*-glucuronide (Api-7-*O*-Gln). Chlorogenic acid (5-CQA, 5-monocaffeoylquinic acid) and 3,5-dicaffeoylquinic acid (3,5-DCQA) are the most abundant quinic acid derivatives, which include also *p*-coumaroylquinic acid (putative assignment). Notably, while main polyphenol and flavonoid derivatives are still detectable, the main components in the LCMS profile in the negative ionization polarity of S are saponins (Figure 1).

Cynarasaponins A/H, E, F/I, and J have been tentatively identified based on accurate mass measurements, fragmentation pattern, and comparison with literature data [29].

### 2.4. (Semi-)Quantitative Determination of Main Polyphenols and Saponins in Cynara cardunculus L. subsp. scolymus Extracts by UHPLC-ESIMS^−^

To provide a quantitative chemical characterization of this cultivar, the amount of the different specialized metabolites was calculated by UHPLC-HRESI-MS/MS in negative ion polarity using the external standard calibration approach. For each polyphenolic metabolite, a calibration curve was built by using the corresponding commercial standard; the quantitative content was inferred in the various tissues and expressed as mg/100 g DW and mg/g extract (Table 2 and Supplementary Material, Table S2). As anticipated by qualitative analysis, the most abundant metabolite in artichoke heads was Api-7-*O*-Gln with 103.95 and 71.51 mg in 100 g DW of H and E, respectively. Together with the other two glycosides, i.e., rutinoside and glucoside, apigenin derivatives account for 132.80 and 78.25 mg/100 g DW of H and E, respectively. On the other hand, the glycoside derivatives of the flavonoid luteolin, including its glucosides, rutinoside, and glucuronide, are less abundant and taken together represent 33.88 and 23.95 mg/100 g DW in H and E, respectively. Chlorogenic acid is the most abundant among the quinic acid derivatives (35.62 and 43.70 mg/100 g DW in H and E extracts, respectively), and it is recovered in a large amount also in stem (153.11 mg/100 g DW). However, the dominant metabolites in the stem are not polyphenols but triterpenoid glucosides, namely the cynarasaponins, especially cynarasaponin A which alone represents about 1% of the stem dry material (based on the amount calculated as escin equivalent).

### 2.5. Qualitative Profile of Anthocyanins in Cynara cardunculus L. subsp. scolymus Extracts by UHPLC-ESIMS^+^

**Figure 2:**
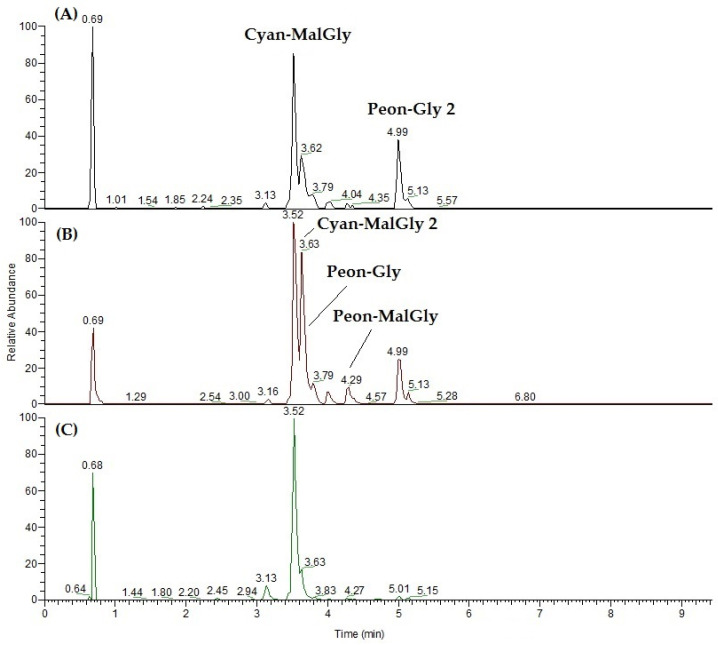
Representative UHPLC-ESIMS profiles in positive ionization mode (XIC—chromatograms by extracted [M]^+^ ion) of the hydroalcoholic extract of artichoke parts: (**A**) heart with inner bracts (H); (**B**) external bracts (E); and (**C**) stem (S). Cyan-MalGly, Cyanidin malonylglycoside; Cyan-MalGly2, Cyanidin-malonylglycoside 2; Peon-Gly, Peonidin glycoside; Peon-Gly 2, Peonidin glycoside 2; Peon-MalGly, Peonidin malonylglycoside.

The same samples as in Section 2.3 were analysed in duplicate in positive ionization polarity, and the LCMS profiles of the three artichoke sections were compared. Main peaks have been tentatively assigned based on the literature [30] and HR-ESIMS/MS data. Anthocyanins in the H fractions were mainly cyanidin malonylglycosides and peonidin glycosides (Figure 2) while in E the anthocyanin pool also included peonidin malonylglycoside. In S it was mainly detected cyanidin malonylglycoside.

### 2.6. Semi-Quantitative Determination of Main Anthocyanins in Cynara cardunculus L. subsp. scolymus Extracts by UHPLC-ESIMS^+^

Semi-quantitative analysis of anthocyanins was carried out by using an external standard calibration approach based on cyanidin-3-glucoside. The richest source of these pigments came from the external bracts (Table 3 and Supplementary Material, Table S3), where the two isomers of cyanidin malonylglycoside accounted for 70% of the anthocyanin amount (4.64 mg eq cyanidin-3-glucoside/100 g DW).

### 2.7. Antioxidant Activity Assays

#### 2.7.1. DPPH Assay

Hydroalcoholic extracts of the different parts of artichoke (H, E, and S) have been tested for their antioxidant activity by using a DPPH assay, which is the easiest method to evaluate the radical scavenging activity. The values were expressed as mg eq TROLOX/g extract (Supplementary Material, Table S1) and next normalized as g eq TROLOX/100 g DW. Extracts of E showed higher antioxidant activity (5.88 ± 0.04 g/100 g DW) than H (3.01 ± 0.01 g/100 g DW) and S (2.66 ± 0.01 g/100 g DW) extracts. (Table 1). The greatest antioxidant activity of E was also confirmed by the IC_50_ value (37.34 ± 0.54 μg/mL), followed by S (69.23 ± 1.24 μg/mL) and H (73.89 ± 2.61 μg/mL) (Table 1).

#### 2.7.2. FRAP Test

The FRAP assay, based on the reduction of Fe^3+^ to Fe^2+^, directly measures the antioxidant or reducing power of a solution. The extracts of individual sections of the plant showed comparable Ferric reducing power activity, in the range 1.8–2.1 mmol Fe^2+^ eq/100 g DW (Table 1) (61–67 μmol Fe^2+^ eq/g extract, Supplementary Material Table S1).

### 2.8. Determination of Cytotoxicity by MTT Assay

**Figure 3:**
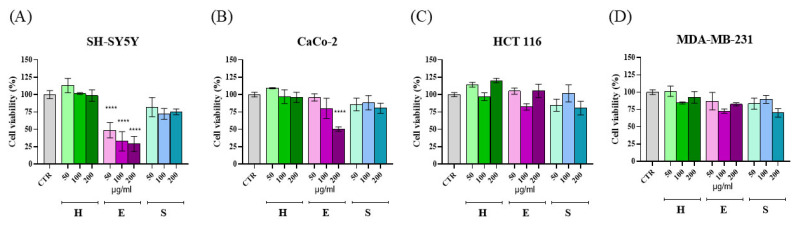
MTT assay for cytotoxicity determination: bar plot representing cell viability (%) of cells treated with different concentrations of artichoke extract (50, 100, and 200 µg/mL) after 24 h. H = heart with inner bracts; E = external bracts; S = stem. (**A**) SH-SY5Y cell line; (**B**) CaCo-2 cell line; (**C**) HCT 116 cell line; (**D**) MDA-MB-231 cell line. Statistical analysis was performed using ANOVA for multiple comparisons (**** *p* < 0.001).

The viabilities of human neuroblastoma SH-SY5Y, colorectal adenocarcinoma CaCo-2, large intestine colon carcinoma HCT 116, and breast adenocarcinoma MDA-MB-231 cells were measured at 24 h after treatment with H, E, and S extracts at three different dilutions (50, 100, and 200 μg/mL) prepared as reported in Section 4.8.1 and dissolved in milliQ water, by using the MTT assay (Figure 3). As shown, at 24 h a dose-response reduction in cell viability was observed on SH-SY5Y (Figure 3A) and CaCo-2 cell lines (Figure 3B) treated with extracts from E, with significant activity at 50 μg/mL (*p* < 0.001) and 200 μg/mL (*p* < 0.001), respectively. Conversely, no cytotoxic effect was observed on the other two tumoral cell lines (Figure 3C,D). On HaCaT keratinocytes used as a control cell line, no significant toxicity was observed after treatment with the highest concentration of all the extracts (Supplementary Material, Figure S1).

## 3. Discussion

Here we report a phytochemical and biological characterization of “Carciofo di Procida” collected during the spring season in 2023. We adopted a sustainable EtOH/H_2_O extraction procedure, by using both an ultrasound step and a shaking step at a mild temperature (38 °C) to promote the release of the metabolites from the plant tissues. A comparison with literature data revealed overall a superimposable profile of polyphenol composition although with lower complexity due to the absence of isomeric compounds as reported elsewhere [1,3,18,19,22,31,32]: in fact, within the family of caffeoyl quinic acids only chlorogenic acid and the 3,5-dicaffeoyl isomer were detected as representatives of mono and di-caffeoyl quinic acid derivatives, respectively. From a quantitative standpoint, chlorogenic acids do not represent the dominant compounds in heart and bracts as reported in some cultivars [22,26,33,34]. In fact, in the cultivar from Procida the flavonoid apigenin-7-*O*-glucuronide was the main component in head sections, in line with the specific phenolic content reported in various cultivars of Romanesco genotype such as ‘Clone C3’ and ‘Tondo di Paestum’ [1,18,19]. Conversely, the extracts of stems were dominated by cynarasaponins. Due to the absence of commercial standards for cynarasaponins, we referred to escin, reported in the literature as reference standard for total saponins content, as a surrogate standard for quantitative analysis [35]. With the approximation being based on this semi-quantitative approach, cynarasaponins accounted for the 1.5% of the DW in stems. These triterpenoids have been reported in the literature to possess an anti-carcinogenesis activity in mouse skin and to inhibit tumor promoter-induced inflammation in mice [36,37]. In addition, cynarasaponins were found to repress the genotoxicity induced by acridine orange and ofloxacin, protecting the chloroplast DNA from the damage induced by these mutagens [38]. Hence, we tested the hydroalcoholic extracts from the stems together with those from the head parts of the artichoke against four human tumor cell lines, including the neuroblastoma cell line SH-SY5Y, the colorectal adenocarcinoma CaCo-2, the breast mammary gland adenocarcinoma MDA-MB-231, and the large intestine colon carcinoma HCT 116 cell lines, at different concentrations (50, 100, and 200 μg/mL). Indeed, we did not detect any significant reduction in cell viability in these tumoral lines treated with extracts S (containing cynarasaponins) and H (particularly enriched in flavonoid glycosides); on the other hand, we observed a clear reduction in viability when these cells were treated with extracts from the external bracts (E), particularly on SH-SY5Y cells at 50 μg/mL (Figure 3). Considering the substantial similarity in terms of the quali-quantitative content of polyphenols in extracts from H and E, we deduced that other components may act eventually in combination with these compounds. In effect, mono- and dicaffeoyl quinic acids, also referred to as ‘chlorogenic acids’, have been reported in the literature to exhibit some cytotoxic properties [39,40,41]. In particular, caffeoyl quinic acids, or extracts containing them, have been described to induce cytotoxicity in human oral tumor HSC-2 and HSG [42], human leukemic HL-60 and Jurkat [43], colorectal adenocarcinoma HT-29 [44], breast cancer MDA-MB-231 and MCF-7 [45,46], and human lung adenocarcinoma A549 and hepatocellular carcinoma HepG2 [47] cell lines, to mention a few. Notably, in contrast with these results, other studies reported the absence of cytotoxicity when these compounds were tested in a pure form, and not as natural extracts or mixtures, against a panel of breast cancer cell lines [48], suggesting that other components in the bioactive extracts or synergic actions may take place to explain the cytotoxic effect as reported in the literature.

Flavonoids have been also reported to exert cytotoxic activity. For example, apigenin-7-*O*-glucuronide (methyl ester) has been reported to show cytotoxic effects on MCF-7 breast cancer in a dose-dependent manner [49], and apigenin-7-*O*-glucoside showed toxicity against HeLa cervical cancer cells [50].

Cytotoxic activity has been reported for extracts from a few artichoke varieties [51,52,53,54,55,56], including several genotypes from Italian regions [11,45,57,58,59,60,61]. While the polyphenol composition may vary, among cultivars and artichoke parts, these studies have elucidated the capacity of various extracts to induce apoptosis in different cell lines (e.g., hepatocellular carcinoma [60], colon cancer [11], neuroblastoma [58], breast cancer [45,57,59], and multiple myeloma [61] cell lines). These actions are attributed to diverse pathways such as the induction of cell cycle arrest, caspase-dependent apoptosis via mitochondrial disruption and altered Bax/Bcl2 ratios (leading to DNA fragmentation), and the modulation of crucial signaling pathways associated with inflammation and proliferation [57,60]. In addition, according to numerous studies, the antioxidant properties exerted by bioactive polyphenols like flavonoids and caffeoylquinic acids, among others, are crucial in preventing cancer [62]. Indeed, the antioxidant activities of artichoke phenolics, due to their H-donating hydroxyl groups, are multifaceted, virtually stemming from their ability to inhibit lipid peroxidation, act as free radical scavengers, chelate metal ions such as iron, and intercepting reactive-oxygen species (ROSs), whose release is tightly linked to cancer development and progression [63].

The antioxidant potential of the extracts from “Carciofo di Procida” was therefore evaluated. By means of DPPH assay (Table 1) the activity of the H extract (IC_50_ = 73.89 µg/mL) resulted comparable with that exhibited by hydroalcoholic extract of heads of “Carciofo di Paestum”, likely the best known variety of Campania region (Italy), which showed an IC_50_ value of 80.51 µg/mL [22]. Compared to this, notably, the antioxidant activity of external bracts of Procida cultivar was even higher, showing an IC_50_ value of 37.34 μg/mL. The highest DPPH antioxidant activity of extracts, from external bracts towards edible parts, was also documented in other studies and positively correlated with cytotoxic effects [64,65].

The antioxidant activity was also measured by using the FRAP assay which generally shows a strong correlation with total phenolic contents [66,67]. The sections of “Carciofo di Procida” showed comparable values when tested for FRAP potential, in line with other values reported in the literature for other cultivars including “Tondo di Paestum”, “Violetto di Sicilia”, “Tempo F1”, and “Tema 2000” [68].

Despite the coherent results for FRAP potential, the remarkable differences observed in radical scavenging (DPPH) and MTT cytotoxicity tests of H, E, and S of “Carciofo di Procida” seemed substantially not ascribable to dissimilarities of chemical profiling of main caffeoyl quinic acids, flavonoids, and/or saponins. However, statistical correlation analyses revealed a significant relationship (Spearman R > 0.6) between cytotoxic activity and high concentration of luteolin-7-*O*-glucoside (Supplementary Material, Table S4).

Prompted by these results and with the aim to perform a study as comprehensive as possible of the phenolic composition of this cultivar, we decided to investigate also the specific anthocyanin content and profile in the various artichoke parts.

Anthocyanins are flavonoid plant pigments responsible for the brilliant color (red to blue) of leaves, flowers, and fruits. They encompass several glycosidic derivatives of anthocyanidins, including cyanidin, delphinidin, pelargonidin, peonidin, petunidin, and malvidin. Among polyphenols, they represent an important group of antioxidant molecules. Furthermore, their multiple beneficial health effects have been associated to anti-inflammatory, chemo-protecting and antiproliferative properties as well as to neuroprotective activity, as evaluated in in vitro and in vivo studies [69,70,71,72,73]. Peonidin-3-*O*-glucoside inhibits lung cancer metastasis [74], and interestingly, some studies evidenced the additive effects in association with lutein on CaCo-2 cells [75]. Peonidin-3-*O*-glucoside and cyanidin-3-*O*-glucoside displayed strong inhibitory effects on the cell growth of highly metastatic breast cancer and suppressed tumor growth in vivo, in combination with chemotherapy agents such as doxorubicin [76]. Hence, the importance of anthocyanins as nutraceutical ingredients is scientifically supported, and their application in cancer chemoprevention is promising.

Very few studies report the anthocyanin composition in edible artichoke or its by-products. The most recent by Schutz [30] described anthocyanins in selected cultivars by means of LCMS techniques, identifying as the main component cyanidin-3-(6″-malonylglucoside), followed by cyanidin-3-glucoside, cyanidin-3,5-malonyldiglucoside, and cyanidin-3-(3″-malonylglucoside), plus a series of minor cyanidin-, peonidin-, and delphinidin-glycosides.

Indeed, by running LCMS analysis we could not detect in our extracts cyanidin-3-glucoside or cyanidin diglycosides. On the other hand, we found that two isomers of cyanidin malonylglycosides were the most abundant anthocyanins (amounts reported as cyanidin-3-glucoside equivalents) in external leaves, co-occurring with two isomers of peonidin glycoside and a peonidin malonylglycoside. The variability of anthocyanins within artichoke varieties, in terms of both their total content and relative proportions, has been previously evidenced [30]. This is consistent with the broader genotype/ecotype chemodiversity observed for other specialized metabolites.

Overall, among the three artichoke tissue subsamples, the external part was revealed to be the richest source of these pigments on a semi-quantitative basis (Table 3).

**Figure 4:**
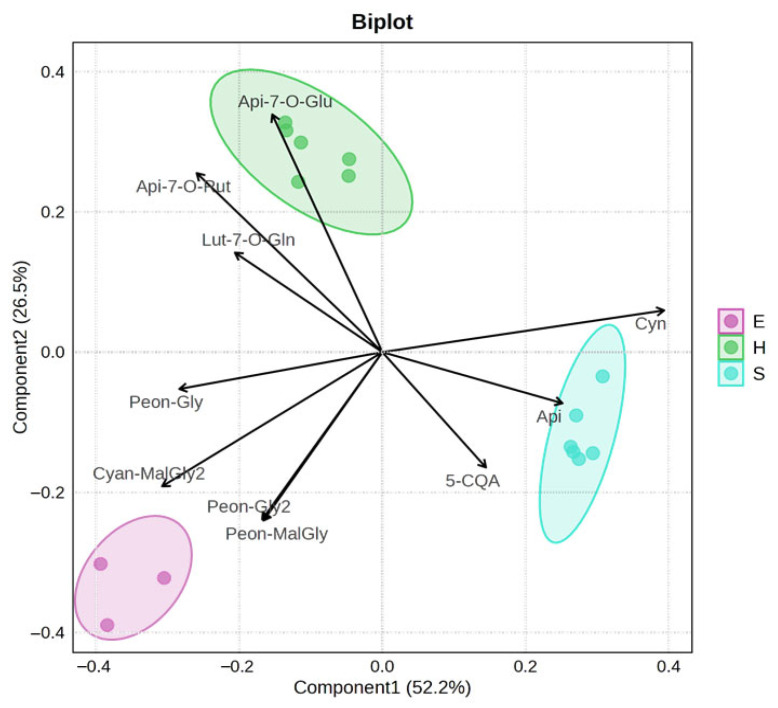
PLS-DA biplot showing the separation of the three artichoke parts (H, E, and S) based on their metabolic profiles. The clear distinction among the groups indicates significant metabolic differences, with the separation of group E primarily driven by anthocyanins.

The PLS-DA biplot (Figure 4), used to identify the metabolites contributing to the differentiation among artichoke parts, revealed a clear separation among the three groups (H, E, and S), indicating distinct metabolic profiles. Notably, anthocyanins emerged as key discriminant compounds, particularly peonidin derivatives and an isomer of cyanidin malonylglycoside, which were more abundant in E. Furthermore, statistical correlation analyses revealed a significant relationship (Spearman R > 0.6) between cytotoxic activity and all the identified anthocyanins (Supplementary Material, Table S4).

## 4. Materials and Methods

### 4.1. Chemicals

Commercial standards of 5-monocaffeoylquinic acid (chlorogenic acid) [CAS 327-97-9], 3,5-dicaffeoylquinic acid [CAS 2450-53-5], luteolin, apigenin, luteolin-7-*O*-rutinoside, luteolin-7-*O*-glucoside, apigenin-7-*O*-glucoside, escin, 2.2-diphenyl-1-picrylhydrazyl (DPPH), Trolox, quercetin, and Folin–Ciocalteau’s phenol reagent were purchased by Merck, Milan, Italy; apigenin-7-*O*-rutinoside, apigenin-7-*O*-glucuronide, and luteolin-7-*O*-glucuronide were from Vetrochimica (Casandrino (NA), Italy). Ultrapure water for LCMS was obtained by a MilliQ apparatus (Merck, Milan, Italy). Methanol and Acetonitrile (ACN) were LCMS grade; all the other solvents were HPLC-grade. All solvents have been purchased from VWR (Milan, Italy).

### 4.2. Biological Material, Artichoke Collection and Sample Preparation

The artichoke cultivar “Carciofo di Procida” (*Cynara cardunculus* L. subsp. *scolymus*) was cultivated in the spring of 2023 in a home-producing field in Procida, an island of the Gulf of Naples (Italy), in Solchiaro area (40°44′37″ N. 14°00′38″ E. 36 mt asl). In May 2023, artichokes at commercial and edible maturity were harvested, washed with tap water, and manually cleaned. Successively, external bracts, hearts with inner bracts, and stems were separated, stored frozen at −20 °C, and freeze-dried. For hearts and stems, six random samples were selected and extracted in technical replicate. External bracts were combined and extracted in triplicate.

### 4.3. Total Polyphenol Content (TPC)

Lyophilized material (60 mg) from six H and six S (in duplicate) and pooled E subsamples (in triplicate) were extracted with EtOH:H_2_O (1:1) (6 mL × 2) under shaking for 1 h at 38 °C at 100 rpm. The extraction solvent mixture was separated from the solid residue by centrifugation at 10,000× *g* for 10 min at 10 °C (Avanti^TM^ J-25, Beckman Coulter, Milan, Italy); the pooled liquid phases were dried under nitrogen stream, lyophilized, and stored at −20 °C until analysis.

The total polyphenol content was estimated by using the Folin–Ciocalteau method. Hydroalcoholic extracts (ranging from 10 to 50 μL with a concentration of 20 mg/mL), 800 μL of deionized water, and 50 μL of Folin–Ciocalteau’s phenol reagent were accurately mixed. After 1 min, 100 μL of a 20% sodium carbonate solution was added and further mixed. A final volume of 1 mL was reached by adding deionized water. Quercetin was used as standard. Samples were kept at room temperature for 2 h, and then the total phenol content was estimated by reading at λ 765 nm (Genesys 150, Thermo Fisher Scientific, Waltham, MA, USA). TPC was expressed as mg/g DW and mg/g extract.

### 4.4. Total Monomeric Anthocyanin Content (TAC)

As above reported, lyophilized samples of the different tissues (50 mg) were transferred into a 1.5-mL microcentrifuge tube and extracted with 1 mL of MeOH/H_2_O/TFA (70:30:0.5, *v*/*v*/*v*) in an orbital shaker (TS-100C, Biosan, Riga, Latvia) at 100 rpm for 1 h at room temperature according to the protocol reported by Barnes and colleagues [77]. The samples were successively sonicated for 20 min and centrifuged at 10,000× *g* for 10 min at 10 °C using a microcentrifuge (MicroStar 17R, VWR International, Milan, Italy). The supernatant was filtered through paper and used for the monomeric anthocyanin content assay (TAC) according to [28]. Briefly, 500 µL of each sample were diluted 1:2 with buffer solutions at pH 1.0 (hydrochloric acid-potassium chloride) and pH 4.5 (hydrochloric acid-sodium acetate). Samples were incubated at 25 °C in the dark for 15 min, and then the spectrophotometric absorbance was measured at λ = 510 and 700 nm. AC was expressed as mg cyanidin-3-glucoside equivalents using the following equation:

AC = A × MW × DF × 1000/ε × *l*

where A = (A_510_ − A_700_)_pH1.0_ − (A_510_ − A_700_)_pH4.5_, MW is the molecular weight of the cyanidin-3-glucoside (445.2 g mol^−1^), DF is the dilution factor, ε is the molar extinction coefficient of cyanidin-3-glucoside (26,900 L cm^−1^ mol^−1^), and *l* is the pathlength in cm.

### 4.5. Free Radical-Scavenging Assay (DPPH Assay)

The free radical-scavenging ability of hydroalcoholic extracts was evaluated by means of the DPPH (2,2-diphenyl-1-picrylhydrazyl free radical) method [78,79] and expressed as mg equivalent of Trolox, the water-soluble analogue of vitamin E [80]. It was used as a standard for comparing the free radical scavenging activity of samples expressed as Trolox equivalent antioxidant capacity (TEAC) [81]. Specifically, 50 µL of sample solution (prepared as reported in Section 4.3 at a concentration of 20 mg/mL) was added to 0.7 mL of DPPH in methanol (0.1 mM final concentration) and adjusted to a final volume of 2 mL with methanol. The absorbance was determined after 30 min at λ = 517 nm at room temperature, and the percentage of free radical inhibition was calculated. TEAC values were calculated by using the calibration curve built with different amounts of standard Trolox (from 5 μg up to 20 μg).

### 4.6. Ferric Reducing Power Activity (FRAP Assay)

The ferric reducing antioxidant power of hydroalcoholic extracts was estimated by means of a FRAP assay [82]. This assay is based on the ability to reduce a yellow ferric complex [Fe^3+^-TPTZ (2.4.6-tripyridyl-s-triazine)] to a blue ferrous complex (containing Fe^2+^) by electron-donating antioxidants in an acidic medium. The FRAP Assay kit (Cat. N. MAK369, Merck, Milan, Italy) was used according to the manufacturer’s instructions. Hydroalcoholic extracts prepared as reported in Section 4.3 were dissolved in methanol at a concentration of 20 mg/mL, and 10 μL was used in each well.

### 4.7. Cytotoxicity Test (MTT Assay)

#### 4.7.1. Cell Cultures

The human colorectal adenocarcinoma CaCo-2 cell line (ATCC HTB-37), the human neuroblastoma cell line SH-SY5Y (ATCC CRL-2266), the human breast mammary gland adenocarcinoma cell line MDA-MB-231 (ATCC HTB-26), and the human large intestine colon carcinoma cell line HCT 116 (ATCC CCL-247) were purchased from the ATCC cell bank (Manassas, VA, USA). The human epidermal keratinocyte HaCaT (item number: 300493; mycoplasma-specific polymerase chain reaction: negative) were primary cells immortalized and were purchased from CLS Cell Lines Service (Eppelheim, Germany).

CaCo-2 cells were grown in DMEM medium supplemented with glutamine 4 mM and high glucose, 10% FBS, 1% penicillin-streptomycin, and 1% non-essential amino acids (Aurogene Srl, Rome, Italy). SH-SY5Y cells were grown in DMEM medium supplemented with glutamine 4 mM and high glucose, 20% FBS, and 1% penicillin-streptomycin (Aurogene Srl, Rome, Italy). HaCaT cells were grown in DMEM medium supplemented with glutamine 1 mM and high glucose, 10% FBS, and 1% penicillin-streptomycin (Aurogene Srl, Rome, Italy). MDA-MB-231 cells were grown in RPMI medium with glutamine, 10% FBS, and 1% penicillin-streptomycin (Aurogene Srl, Rome, Italy). HCT 116 cells were grown in DMEM medium supplemented with glutamine, 10% FBS, and 1% penicillin-streptomycin (Aurogene Srl, Rome, Italy).

#### 4.7.2. MTT Assay

The MTT (3-(4,5-dimethylthiazol-2-yl)-2,5-diphenyltetrazolium bromide) (Merck, Milan, Italy) assay was used to measure the inhibition of viability following cell treatments with a water solution of the hydroalcoholic extracts at 50, 100, and 200 µg/mL in 48-well plates. MTT was added and incubated for three hours at 5% CO_2_ and 37 °C. After this time, the formazan precipitate was dissolved in 200 µL of isopropyl alcohol, and then, the absorbance was measured on a GloMax Explorer Microplate reader (Promega Italia, Milan, Italy) at 600 nm.

### 4.8. Chemical Profiling by UHPLC-ESIMS/MS

#### 4.8.1. Biological Material Extraction

Six individual H and S subsamples (5 mg) and the pooled E were prepared as reported in Section 4.2 and extracted with EtOH/H_2_O (1:1) (0.5 mL × 2) in an orbital shaker (TS-100C, Biosan, Riga, Latvia) set at 900 rpm and 38 °C for 1 h. Extraction solvent mixture was separated from the solid residue by centrifugation at 10,000× *g* for 10 min at 10 °C; the pooled liquid phases were dried under a nitrogen stream, lyophilized, and stored at −20 °C until analysis.

#### 4.8.2. UHPLC-MS/MS Analysis of Polyphenols and Saponins

The LCMS analysis of polyphenols and saponins was performed according to Yahia et al. [83] with some modifications. Briefly, chromatographic separation was obtained by a column Omega Polar C18 (100 × 2.1 mm, 1.6 µm) (Phenomenex, Castel Maggiore (BO), Italy) on an Infinity 1290 UHPLC System (Agilent, Milan, Italy). Elution solvents included the following: (A) water + 0.1% formic acid (FA) and (B) ACN + 0.1% FA. Gradients included the following: 0–1 min, 10% to 20% B; 1–8 min, 20% to 50% B; 8–8.5 min up to 100% B; 8.5–10 min holding 100% B; and then in 1 min a return to initial condition and equilibration for 2 min. The flow was set to 0.4 mL/min. The UHPLC system was coupled to a Q Exactive Mass Spectrometer (Thermo Scientific, San Jose, CA, USA) equipped with a HESI source operating in negative ionization mode. The spectra were acquired over the range 133–2000 *m*/*z*. Optimum values were as follows: spray voltage, 3 kV; capillary temperature, 320 °C; S-lens RF level 60; aux gas heater temperature, 320 °C; sheath gas flow rate, 50; aux gas flow rate, 30; and resolution in full scan 70000. MS/MS experiments were performed with (N)CE at 20, 30, and 40. Resolution in MS/MS mode was set to 17,500. MS data were processed by Xcalibur Software (vers. 3.0.63, San Jose, CA, USA). Plant extracts were suspended in 500 µL of MeOH/H_2_O (1:1) and centrifuged at 10,000× *g* for 10 min at 10 °C: the supernatant, transferred in an autosampler vial, was analyzed in duplicate. The injection volume was 5 µL.

For quantitative purposes, six calibration solutions in the range 30–10,000 ng/mL were prepared by serial dilution of a stock solution of 1 mg/mL in MeOH/H_2_O (1:1) containing the following standards: 5-monocaffeoylquinic acid, 3,5-dicaffeoylquinic acid, luteolin, apigenin, luteolin-7-*O*-rutinoside, luteolin-7-*O*-glucoside, luteolin-7-*O*-glucuronide, apigenin-7-*O*-rutinoside, apigenin-7-*O*-glucoside, apigenin-7-*O*-glucuronide, and escin. Calibration curves were prepared in triplicate. Data were plotted considering as the analytical response the peak area of each polyphenol standard against concentration. The peak area was measured on the extracted ion chromatogram (XIC) of the molecular ion [M − H]^−^. A least-square linear regression weighting by the reciprocal of the concentration was used to best fit the linearity curve (Supplementary Material, Figure S2). The concentration of the various metabolites was inferred by using the calibration curve of the corresponding standard. Absolute recovery and matrix effects were not assessed. For semi-quantitative analysis of *p*-coumaroylquinic acid was used the calibration curve of 5-monocaffeoylquinic acid, while for cynarasaponins, escin was used as the reference standard and results have been expressed as escin mg equivalents.

#### 4.8.3. UHPLC-MS/MS Analysis of Anthocyanins

The LCMS analysis of anthocyanins was carried out on the same UHPLC-QExactive platform operating in positive ionization mode, by using the same chromatographic gradient and samples. Spectra were acquired in the range 133–2000 *m*/*z*. Optimum values were as follows: spray voltage, 3.2 kV; capillary temperature, 320 °C; S-lens RF level 55; aux gas heater temp., 320 °C; sheath gas flow rate, 50; and aux gas flow rate, 30. Resolution was set to 70,000 in Full Scan analysis. MS/MS experiments were performed with (N)CE at 25, 30 and 35. Resolution in MS/MS mode was set at 17,500. MS data were processed by Xcalibur Software (vers. 3.0.63, San Jose, CA, USA). The injection volume was 5 µL.

To perform a semi-quantitative analysis, five calibration solutions in the range 30–3000 ng/mL were prepared by serial dilution of a stock solution of cyanidin-3-*O*-glucoside standard (1 mg/mL) in MeOH/H_2_O (1:1). Calibration curves were prepared in triplicate considering as the analytical response the peak area of the standard, which was measured on the extracted ion chromatogram (XIC) of the molecular ion M^+^, against concentration. A least-square linear regression weighting by the reciprocal of the concentration was used to best fit the linearity curve (Supplementary Material, Figure S2). The concentration of the various anthocyanins was inferred by using the calibration curve of the above standard assuming a similar response, due to the chemical similarity, and expressed as cyanidin-3-*O*-glucoside mg equivalents.

### 4.9. Statistical Analysis

Statistical analysis and data plotting were performed using GraphPad Prism software (vers. 10.3.1, GraphPad Software, San Diego, CA, USA). The differences between groups were evaluated using one-way ANOVA followed by multiple comparison test.

Metabolite data were log-transformed and Pareto-scaled, and differences between artichoke parts were assessed using partial least squares discriminant analysis (PLS-DA). The separation of samples was visualized using a biplot, which was also used to identify key metabolites driving the discrimination, using MetaboAnalyst Version 6.0.

## 5. Conclusions

A characterization of the cultivar “Carciofo di Procida” has been carried out, focusing phytochemical and biological analyses on edible parts and by-products of the food plant. The specific content of caffeoyl quinic acids, flavonoids, saponins, and anthocyanins has been assessed by LCMS analysis of the hydroalcoholic extracts. In addition to antioxidant properties associated with heart, stem, and external bracts, a significant cytotoxic activity against neuroblastoma SH-SY5Y and colorectal adenocarcinoma CaCo-2 cell lines was associated mainly to external bracts of the immature inflorescence. A comparative chemical study supported by statistical analysis pointed towards the anthocyanins as putative metabolite discriminants, due to their specific occurrence in the purple tissues, thus supporting these pigments as the components driving the observed bioactivity. However, the nutraceutical potential of agri-food products, exhibiting a wealth of health-promoting effects for humans, likely rely on the contribution of the whole, complex mixture of phytochemicals they contain. These compounds may act in a synergistic manner, and the beneficial properties may not be directly ascribable to a specific bioactive compound or class of compounds. In principle, we cannot exclude also the involvement of other bioactive molecules, such as terpenoids and other lipid derivatives, that were not included in our study. Further investigations are needed to isolate and test the bioactivity of individual components, alone or in combination, to disclose the molecular basis of the cytotoxic effect.

On the other hand, the interesting outcome is that the cytotoxic bioactivity toward selected human cancer cell lines was associated with extracts from a part of the plant (the external bracts) that is regarded as a waste, usually discarded in both home and industrial manipulations to obtain the edible inflorescence. Overall, this study strengthens the basis of recovery and valorization of artichoke by-products in a circular economy perspective, particularly in nutraceutical formulations.

## Figures and Tables

**Table 1:** Total polyphenol content (TPC), monomeric anthocyanin content (TAC), and antioxidant activity of artichoke extracts of heart with inner bracts (H), external bracts (E) and stems (S). Values are expressed as mean ± standard deviation. Different letters (a, b, c) indicate statistically significant differences at *p* < 0.05 (one-way ANOVA).

	TPC	TAC	DPPH	DPPH	FRAP
	mg/g DW	mg/100 g DW	g eq TROLOX/	IC_50_ μg/mL	μmol Fe^2+^ eq/
			100 g DW		100 g DW
H	1.20 ± 0.06 ^b^	1.50 ± 0.04 ^b^	3.01 ± 0.01 ^b^	73.89 ± 2.61 ^a^	2118.44 ± 0.90 ^a^
E	3.40 ± 0.20 ^a^	12.70 ± 0.02 ^a^	5.88 ± 0.04 ^a^	37.34 ± 0.54 ^c^	1802.28 ± 1.07 ^c^
S	0.75 ± 0.04 ^c^	-	2.66 ± 0.01 ^c^	69.23 ± 1.24 ^b^	1890.38 ± 1.56 ^b^

**Table 2:** (Semi-)Quantitative amounts of polyphenols and saponins measured as [M−H]^−^ ions in H (heart with inner bracts), E (external bracts), and S (proximal stem) extracts of artichoke cultivar “Carciofo di Procida”. Results are reported as mg/100 g dry weight (DW), mean values ± standard deviation (SD). LOQ = limit of quantitation, referred to the lowest point of the calibration curve at 30 ng/mL. Luteolin was detectable but always below the LOQ. Different letters (a, b, c) indicate statistically significant differences at *p* < 0.05 (one-way ANOVA) among H, E, and S.

Compound					H		E		S	
	Exact Mass	Measured *m*/*z*	MS/MS	Rt	mg/100 g	SD	mg/100 g	SD	mg/100 g	SD
					DW		DW		DW	
Chlorogenic acid *	353.0873	353.0888	191.0556	1.85	35.62 ^b^	3.67	43.7 ^b^	1.90	153.11 ^a^	59.71
*p*-Coumaroyl quinic acid	337.0934	337.0938	191.0556	2.24	31.59 ^a^	3.57	8.94 ^c^	0.43	15.03 ^b^	3.30
3,5-Dicaffeoyl quinic acid *	515.1195	515.1204	353.0882/191.0556	3.23	5.84 ^b^	1.26	9.04 ^b^	0.66	31.91 ^a^	15.69
Luteolin-7-*O*-rutinoside	593.1506	593.1522	285.0408	2.78	0.30 ^b^	0.09	1.16 ^a^	0.09	1.74 ^a^	0.84
Luteolin-7-*O*-glucuronide	461.0720	461.0735	285.0409	2.96	31.39 ^a^	3.46	19.03 ^b^	0.52	4.45 ^c^	0.89
Luteolin-7-*O*-glucoside	447.0933	447.0938	285.0409	2.98	2.19	0.46	3.76	0.30	2.70	1.30
Apigenin-7-*O*-rutinoside	577.1563	577.1570	269.0460	3.26	8.04 ^a^	1.50	3.35 ^b^	0.17	0.25 ^c^	0.02
Apigenin-7-*O*-glucoside	431.0978	431.0987	269.0460	3.49	20.80 ^a^	2.31	3.38 ^b^	0.17	1.42 ^b^	0.19
Apigenin-7-*O*-glucuronide	445.0776	445.0782	269.0460	3.52	103.95 ^a^	5.00	71.52 ^b^	2.74	45.82 ^c^	3.67
Apigenin	269.0455	269.0458	151.0026/117.0332	5.72	1.72 ^b^	0.38	1.10 ^b^	0.40	13.14 ^a^	3.30
Cynarasaponin J ^#^	941.4752	941.4767	779.4255/629.3701/471.3501	5.71	11.64 ^b^	3.56	<LOQ		193.68 ^a^	45.59
Cynarasaponin F/I ^#^	779.4223	779.4237	717.4263/629.3701/471.3501	5.95	17.48 ^b^	4.46	<LOQ		108.21 ^a^	27.59
Cynarasaponin E ^#^	809.4329	809.4336	647.3750/471.3501	6.11	1.11 ^b^	0.42	<LOQ		69.84 ^a^	24.27
Cynarasaponin A/H ^#,†^	925.4832	925.4818	763.4285/613.3754/455.3527	6.57, 6.84	29.02 ^b^	9.24	2.02 ^b^	0.09	891.90 ^a^	149.11

* Due to confusing nomenclature in the literature, the CAS number has been reported in Section 4.1. ^#^ Data expressed as mg escin equivalents/100 g DW. ^†^ Peak areas were combined for amount calculation.

**Table 3:** Semi-quantitative amounts of main anthocyanins measured as [M]^+^ ions in H (heart with inner bracts), E (external bracts), and S (proximal stem) extracts of artichoke cultivar “Carciofo di Procida”. Results are reported as mg eq cyanidin-3-*O*-glucoside/100 g dry weight (DW), mean values ± SD; nd—not detected. Different letters (a, b) indicate statistically significant differences at *p* < 0.05 (one-way ANOVA) among H, E, and S.

Compound					H		E		S	
	Exact Mass	Measured *m*/*z*	MS/MS	Rt	mg/100 g DW	SD	mg/100 g DW	SD	mg/100 g DW	SD
Cyanidin malonylglycoside	535.1082	535.1088	287.0549	3.52	0.39 ^b^	0.22	2.25 ^a^	0.03	1.98 ^a^	0.97
Cyanidin malonylglycoside 2	535.1082	535.1088	287.0549	3.63	0.10 ^b^	0.08	2.39 ^a^	0.43	nd	
Peonidin glycoside	463.1235	463.1239	301.0708	3.69	nd		0.68	0.33	nd	
Peonidin malonylglycoside	549.1239	549.1250	301.0708	4.29	nd		0.56	0.43	nd	
Peonidin glycoside 2	463.1235	463.1239	301.0708	4.99	0.21 ^b^	0.06	0.85 ^a^	0.09	nd	

## Data Availability

Data are available on request from the corresponding author.

## Supplementary Materials

The following supporting information can be downloaded at https://www.mdpi.com/article/10.3390/molecules30153285/s1: Tables S1–S3: Quantitative data referred to g of extract. Table S4: Spearman’s rank correlation coefficients and *p*-values between cell viability and the concentration of individual metabolites across different cell lines. Figure S1: MTT test on HaCaT cell line. Figure S2: Calibration curves used for the quantitative analyses.
