# Supplementary material for: A Phytochemical and Biological Characterization of Cynara cardunculus L. subsp. scolymus Cultivar “Carciofo di Procida”, a Traditional Italian Agri-Food Product (PAT) of the Campania Region
Source: Molecules. 2025 Aug 5;30(15):3285. doi: 10.3390/molecules30153285 (PMC12348833; doi:10.3390/molecules30153285)
Supplement: Supplementary file 1 [file molecules-30-03285-s001.zip › molecules-3782806-supplementary.pdf]

Supplementary Material

A Phytochemical and biological characterization of *Cynara cardunculus* L. subsp. *scolymus* cultivar "Carciofo di Procida", a Traditional Italian Agri-food Product (PAT) of the Campania Region

Giuseppina Tommonaro[†], Giulia De Simone[†], Carmine Iodice, Marco Allarà, Adele Cutignano[*]

CNR-Istituto di Chimica Biomolecolare, via Campi Flegrei, 34 - 80078 Pozzuoli (Naples), Italy; giuseppina.tommonaro@cnr.it; giulia.desimone@cnr.it; carmine.iodice@cnr.it; marco.allara@cnr.it

* Correspondence: adele.cutignano@cnr.it

† These authors equally contributed.

Content

Table S1: Total polyphenol content (TPC) and antioxidant activity of artichoke extracts of heart with inner bracts (H), external bracts (E) and proximal stems (S), expressed as mg/g extract (EXT).

Table S2: (Semi-)quantitative amounts of polyphenols and saponins measured as $[M-H]^-$ ion in H (Heart with Inner bracts), E (External bracts) and S (Proximal stem) extracts of artichoke cultivar "Carciofo di Procida". Results are reported as mg/g extract (EXT) ± SD. LOQ = limit of quantitation, referred to the lowest point of the calibration curve at 30 ng/ml. Luteolin was detectable but always below the LOQ.

Table S3: Semi-quantitative amounts of main anthocyanins measured as [M]+ ion in H (Heart with Inner bracts), E (External bracts) and S (Proximal stem) extracts of artichoke cultivar "Carciofo di Procida". Results are reported as µg eq cyanidin-3-O-glucoside /g extract (EXT) ± SD; nd-not detected.

Table S4: Spearman's rank correlation coefficients (R) and p-values (*p<0.05; **p<0.01) between cell viability and the concentration of individual metabolites across different cell lines.

Figure S1. MTT assay for cytotoxicity determination: bar plot representing cell viability (%) of HaCaT cell line treated with the highest concentration (200 µg/mL) after 24h. H = heart with inner bracts, E = external bracts, S = stem.

Figure S2: Calibration curve of A) Chlorogenic acid; B) 3,5-dicaffeoyl quinic acid; C) Apigenin; D) Luteolin-7-O-rutinoside; E) Luteolin-7-O-glucoside; F) Luteolin-7-O-glucuronide; G) Apigenin-7-O-rutinoside; H) Apigenin-7-O-glucoside; I) Apigenin-7-O-glucuronide; J) Escin K) Cyanidin-3-glycoside

Table S1: Total polyphenol content (TPC) and antioxidant activity of artichoke extracts of heart with inner bracts (H), external bracts (E) and proximal stems (S), expressed as mg/g extract (EXT).

	TPC	DPPH	FRAP
	**mg/g EXT**	**mg eq TROLOX/g EXT**	**µmol Fe^{2+} eq/ g EXT**
**H**	3.28 ±0.09	87.29 ± 0.04	61.39 ± 2.69
**E**	11.58 ±0.71	218.15 ± 0.20	66.11 ± 1.70
**S**	2.77 ±0.13	93.14 ± 0.05	66.90 ± 0.72

Table S2: semi-quantitative amounts of polyphenols and saponins measured as [M-H]- ion in H (Heart with Inner bracts), E (External bracts) and S (Proximal stem) extracts of artichoke cultivar "Carciofo di Procida". Results are reported as mg/g extract (EXT) ± SD. LOQ = limit of quantitation, referred to the lowest point of the calibration curve at 30 ng/ml. Luteolin was detectable but always below the LOQ.

COMPOUND	Exact mass	*m/z*	Rt	H		E		S	
				**mg/g EXT**	*SD*	**mg/g EXT**	*SD*	**mg/g EXT**	*SD*
**CHLOROGENIC ACID**	353.0873	353.0888	1.85	0.77	*0.08*	1.18	*0.10*	2.96	*0.95*
***p*-COUMAROYL QUINIC ACID**	337.0934	337.0938	2.24	0.68	*0.07*	0.09	*0.00*	0.39	*0.09*
**3.5-DICAFFEOYL QUINIC ACID[a]**	515.1195	515.1204	3.23	0.13	*0.03*	0.25	*0.03*	0.78	*0.04*
**LUTEOLIN-7-O-RUTINOSIDE**	593.1506	593.1522	2.78	0.07	*0.00*	0.03	*0.00*	0.04	*0.01*
**LUTEOLIN-7-O-GLUCURONIDE**	461.072	461.0735	2.96	0.68	*0.08*	0.51	*0.02*	0.11	*0.02*
**LUTEOLIN-7-O-GLUCOSIDE**	447.0933	447.0938	2.98	0.05	*0.01*	0.10	*0.00*	0.07	*0.03*
**APIGENIN-7-O-RUTINOSIDE**	577.1563	577.157	3.26	0.18	*0.03*	0.09	*0.00*	0.06	*0.00*
**APIGENIN-7-O-GLUCOSIDE**	431.0978	431.0987	3.49	0.45	*0.06*	0.09	*0.00*	0.04	*0.00*
**APIGENIN-7-O-GLUCURONIDE**	445.0776	445.0782	3.52	2.25	*0.13*	1.92	*0.09*	1.20	*0.09*
**APIGENIN**	269.0455	269.0458	5.72	0.04	*0.00*	0.03	*0.01*	0.36	*0.10*
**CYNARASAPONIN J [#]**	941.4752	941.4767	5.71	0.26	*0.08*	<LOQ		5.27	*1.47*
**CYNARASAPONIN F/I [#]**	779.4223	779.4237	5.95	0.38	*0.10*	<LOQ		2.96	*0.89*
**CYNARASAPONIN E [#]**	809.4329	809.4336	6.11	0.03	*0.01*	<LOQ		1.94	*0.77*
**CYNARASAPONIN A/H[#,†]**	925.4832	925.4818	6.57, 6.84	0.64	*0.21*	0.05	*0.00*	23.99	*4.92*

[a] Due to confusing nomenclature in the literature, the CAS number has been reported in Material and Methods

[#] Data expressed as mg escin equivalents/g EXT

[†] Peak areas were combined for amount calculation.

Table S3: Semi-quantitative amounts of main anthocyanins measured as $[M]^+$ ion in H (Heart with Inner bracts), E (External bracts) and S (Proximal stem) extracts of artichoke cultivar "Carciofo di Procida". Results are reported as μg eq cyanidin-3-O-glucoside /g extract (EXT) ± SD; nd-not detected.

COMPOUND				H		E		S	
	Exact mass	*m/z*	Rt	μg/g		μg/g		μg/g	
				EXT	*SD*	EXT	*SD*	EXT	*SD*
**CYANIDIN MALONYLGLYCOSIDE**	535,1082	535,1088	3,52	8,69	*4,95*	58,9	*5,53*	52,85	*24,90*
**CYANIDIN MALONYLGLYCOSIDE 2**	535,1082	535,1088	3,63	2,16	*1,71*	62,13	*3,63*	nd	
**PEONIDIN GLYCOSIDE**	463,1235	463,1239	3,69	nd		17,05	*5,61*	nd	
**PEONIDIN MALONILGLYCOSIDE**	549,1239	549,1250	4,29	nd		13,53	*8,38*	nd	
**PEONIDIN GLYCOSIDE 2**	463,1235	463,1239	4,99	4,6	*1,33*	22,22	*1,99*	nd	

Table S4: Spearman's rank correlation coefficients (R) and p-values (*p<0.05; **p<0.01) between cell viability and the concentration of individual metabolites across different cell lines.

**Metabolite Name**	**Cell viability on CaCo-2**	**Cell viability on SY-5H5Y**
	**R (p-value)**	**R (p-value)**
**Chlorogenic acid**	**0.144 (0.454)**	**0.377 (0.377)**
**3,5-dicaffeoyl quinic acid**	**0.094 (0.470)**	**0.329 (0.393)**
***p*-coumaroyl quinic acid**	**0.893 (0.149)**	**0.759 (0.226)**
**Luteolin-7-o-rutinoside**	**-0.416 (0.363)**	**-0.186 (0.440)**
**Luteolin-7-o-glucoside**	**-1.000 (0.002)****	**-0.969 (0.079)**
**Luteolin-7-o-glucuronide**	**0.266 (0.414)**	**0.028 (0.491)**
**Apigenin-7-o-rutinoside**	**0.421 (0.362)**	**0.192 (0.439)**
**Apigenin-7-o-glucoside**	**0.681 (0.262)**	**0.486 (0.339)**
**Apigenin-7-o-glucuronide**	**0.374 (0.378)**	**0.142 (0.455)**
**Apigenin**	**0.250 (0.420)**	**0.474 (0.343)**
**Peonidin glycoside**	**-0.851 (0.176)**	**-0.952 (0.099)**
**Peonidin glycoside2**	**-0.950 (0.101)**	**-0.997 (0.024)***
**Cyanidin malonylglycoside**	**-0.829 (0.189)**	**-0.671 (0.266)**
**Peonidin malonylglycoside**	**-0.950 (0.101)**	**-0.997 (0.024)***
**Cyanidin malonylglycoside2**	**-0.938 (0.113)**	**-0.994 (0.036)***

Figure S1. MTT assay for cytotoxicity determination: bar plot representing cell viability (%) of HaCaT cell line treated with the highest concentration (200 µg/mL) after 24h. H = heart with inner bracts, E = external bracts, S = stem.

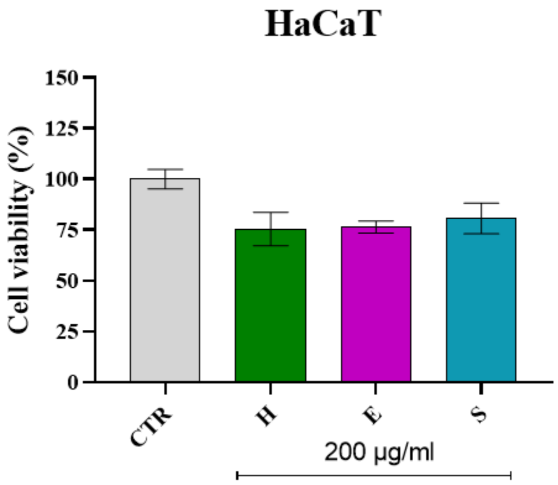

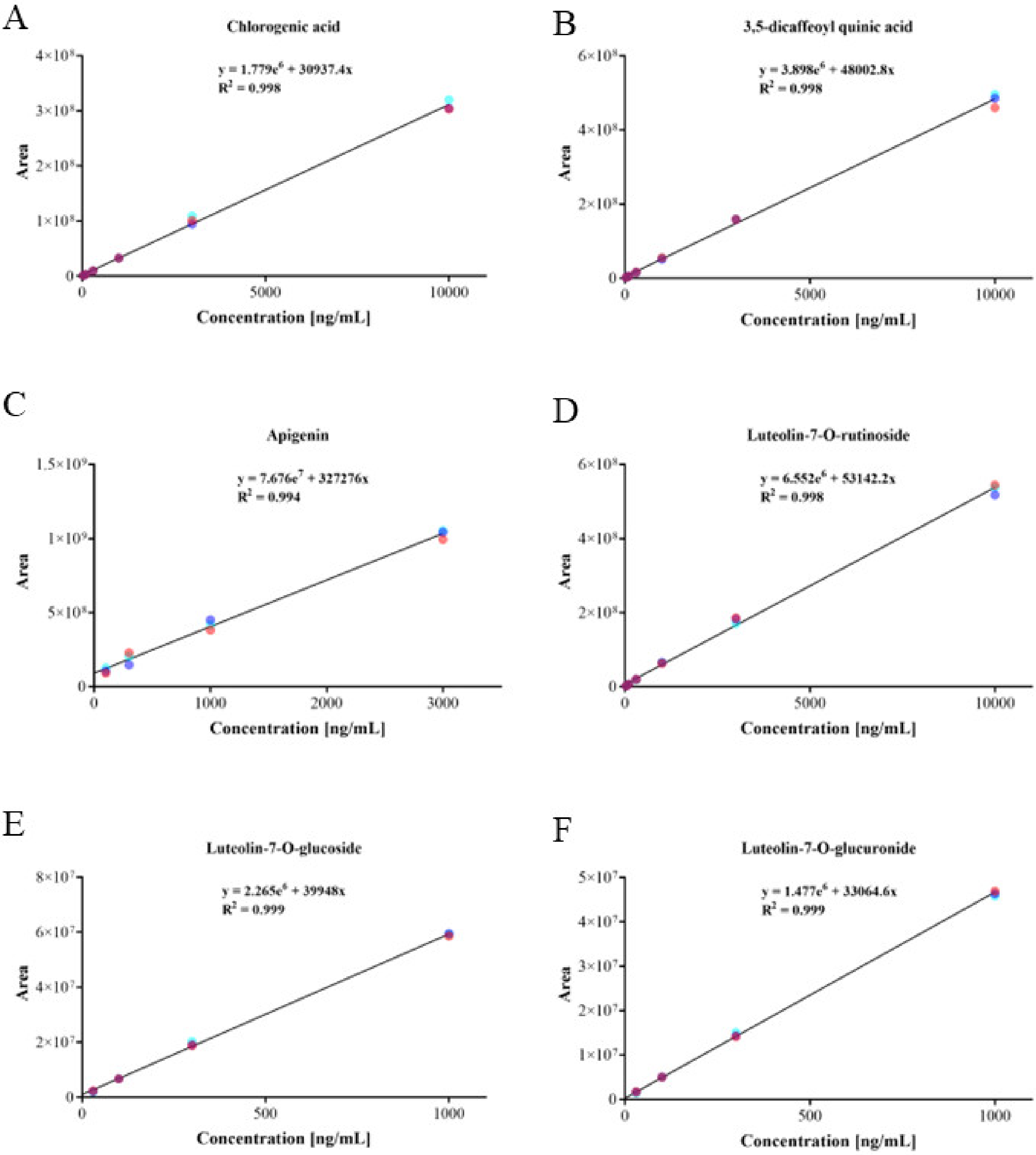
A
Chlorogenic acid
y = 1.779e6 + 30937.4x
R2 = 0.998
Area
Concentration [ng/mL]
B
3,5-dicaffeoyl quinic acid
y = 3.898e6 + 48002.8x
R2 = 0.998
Area
Concentration [ng/mL]
C
Apigenin
y = 7.676e7 + 327276x
R2 = 0.994
Area
Concentration [ng/mL]
D
Luteolin-7-O-rutinoside
y = 6.552e6 + 53142.2x
R2 = 0.998
Area
Concentration [ng/mL]
E
Luteolin-7-O-glucoside
y = 2.265e6 + 39948x
R2 = 0.999
Area
Concentration [ng/mL]
F
Luteolin-7-O-glucuronide
y = 1.477e6 + 33064.6x
R2 = 0.999
Area
Concentration [ng/mL]

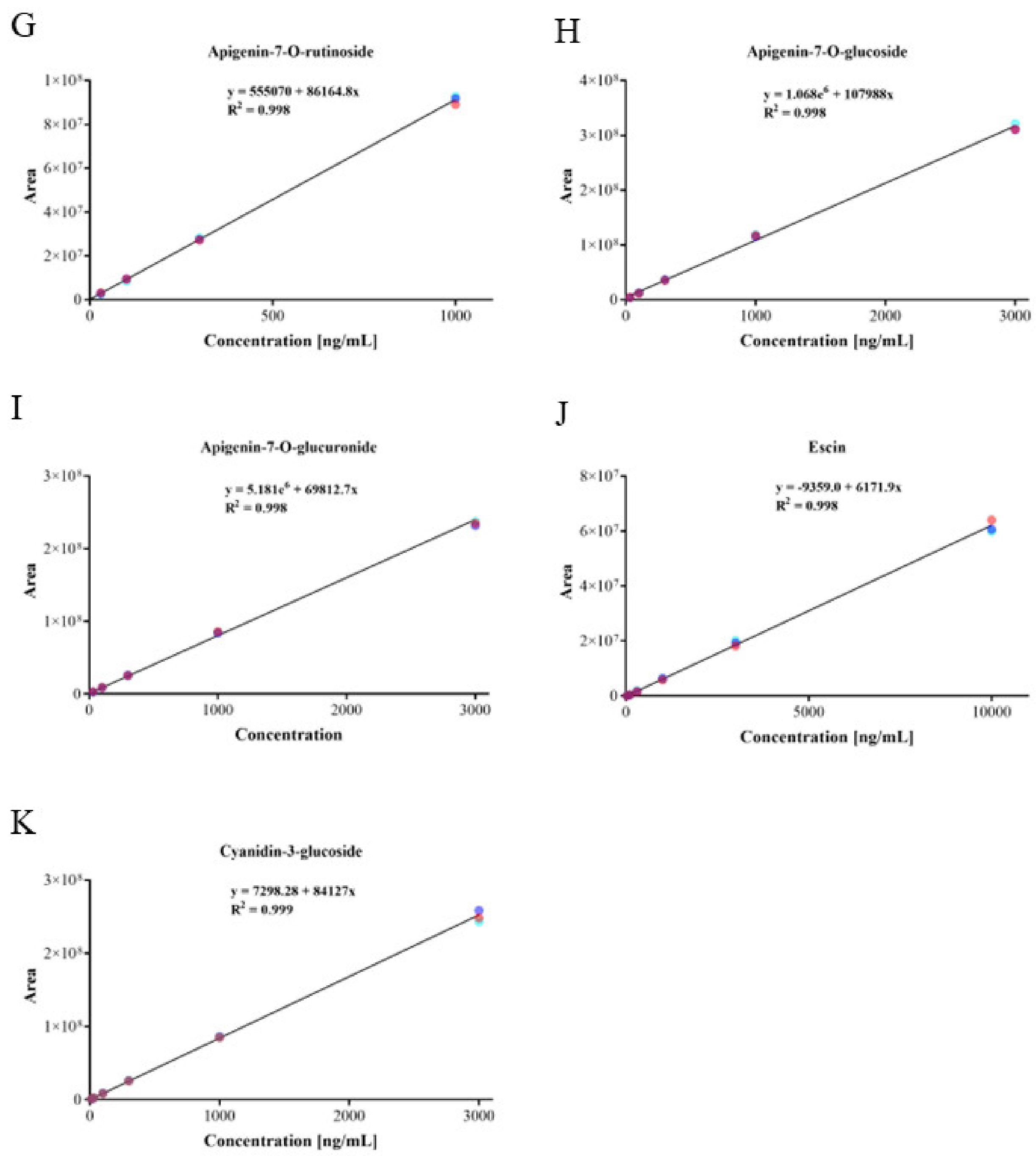

Figure S2: Calibration curve of A) Chlorogenic acid (30-10000 ng/ml); B) 3,5-dicaffeoyl quinic acid (30-10000 ng/ml); C) Apigenin (100-3000 ng/ml); D) Luteolin-7-*O*-rutinoside (30-10000 ng/ml); E) Luteolin-7-O-glucoside (30-1000 ng/ml); F) Luteolin-7-*O*-glucuronide (30-1000 ng/ml); G) Apigenin-7-*O*-rutinoside (30-1000 ng/ml); H) Apigenin-7-*O*-glucoside (30-3000 ng/ml); I) Apigenin-7-*O*-glucuronide (30-3000 ng/ml); J) Escin (30-10000 ng/ml); K) Cyanidin-3-glucoside (30-3000 ng/ml).
